# Quantifying underreporting of law-enforcement-related deaths in United States vital statistics and news-media-based data sources: A capture–recapture analysis

**DOI:** 10.1371/journal.pmed.1002399

**Published:** 2017-10-10

**Authors:** Justin M. Feldman, Sofia Gruskin, Brent A. Coull, Nancy Krieger

**Affiliations:** 1 Department of Social and Behavioral Sciences, Harvard T.H. Chan School of Public Health, Boston, Massachusetts, United States of America; 2 Program on Global Health and Human Rights, Institute for Global Health, University of Southern California, Los Angeles, California, United States of America; 3 Department of Biostatistics, Harvard T.H. Chan School of Public Health, Boston, Massachusetts, United States of America; Massachusetts General Hospital, UNITED STATES

## Abstract

**Background:**

Prior research suggests that United States governmental sources documenting the number of law-enforcement-related deaths (i.e., fatalities due to injuries inflicted by law enforcement officers) undercount these incidents. The National Vital Statistics System (NVSS), administered by the federal government and based on state death certificate data, identifies such deaths by assigning them diagnostic codes corresponding to “legal intervention” in accordance with the International Classification of Diseases–10th Revision (ICD-10). Newer, nongovernmental databases track law-enforcement-related deaths by compiling news media reports and provide an opportunity to assess the magnitude and determinants of suspected NVSS underreporting. Our a priori hypotheses were that underreporting by the NVSS would exceed that by the news media sources, and that underreporting rates would be higher for decedents of color versus white, decedents in lower versus higher income counties, decedents killed by non-firearm (e.g., Taser) versus firearm mechanisms, and deaths recorded by a medical examiner versus coroner.

**Methods and findings:**

We created a new US-wide dataset by matching cases reported in a nongovernmental, news-media-based dataset produced by the newspaper *The Guardian*, The Counted, to identifiable NVSS mortality records for 2015. We conducted 2 main analyses for this cross-sectional study: (1) an estimate of the total number of deaths and the proportion unreported by each source using capture–recapture analysis and (2) an assessment of correlates of underreporting of law-enforcement-related deaths (demographic characteristics of the decedent, mechanism of death, death investigator type [medical examiner versus coroner], county median income, and county urbanicity) in the NVSS using multilevel logistic regression. We estimated that the total number of law-enforcement-related deaths in 2015 was 1,166 (95% CI: 1,153, 1,184). There were 599 deaths reported in The Counted only, 36 reported in the NVSS only, 487 reported in both lists, and an estimated 44 (95% CI: 31, 62) not reported in either source. The NVSS documented 44.9% (95% CI: 44.2%, 45.4%) of the total number of deaths, and The Counted documented 93.1% (95% CI: 91.7%, 94.2%). In a multivariable mixed-effects logistic model that controlled for all individual- and county-level covariates, decedents injured by non-firearm mechanisms had higher odds of underreporting in the NVSS than those injured by firearms (odds ratio [OR]: 68.2; 95% CI: 15.7, 297.5; *p* < 0.01), and underreporting was also more likely outside of the highest-income-quintile counties (OR for the lowest versus highest income quintile: 10.1; 95% CI: 2.4, 42.8; *p* < 0.01). There was no statistically significant difference in the odds of underreporting in the NVSS for deaths certified by coroners compared to medical examiners, and the odds of underreporting did not vary by race/ethnicity. One limitation of our analyses is that we were unable to examine the characteristics of cases that were unreported in The Counted.

**Conclusions:**

The media-based source, The Counted, reported a considerably higher proportion of law-enforcement-related deaths than the NVSS, which failed to report a majority of these incidents. For the NVSS, rates of underreporting were higher in lower income counties and for decedents killed by non-firearm mechanisms. There was no evidence suggesting that underreporting varied by death investigator type (medical examiner versus coroner) or race/ethnicity.

## Abstract 2

In this capture-recapture analysis, Justin Feldman, of Harvard University, and colleagues find that law enforcement-related deaths in the United States are undercounted in official government data derived from state death certificates compared to news media-based data sources.

## Author summary

**Why was this study done?:**

- Several governmental and nongovernmental databases track the number of law-enforcement-related deaths in the US, but all are likely to undercount these deaths.
- To our knowledge, our study is the first to estimate the proportion of law-enforcement-related deaths properly captured by 2 data sources: official US mortality data, derived from death certificates, and The Counted, a nongovernmental database derived from news media reports.
- US mortality data include virtually all deaths that occur in the country, and law-enforcement-related deaths are supposed to be assigned a diagnostic code corresponding to “legal intervention.” If a death is improperly assigned another code, it is considered to be misclassified, which leads to undercounting of the number of law-enforcement-related deaths. We investigated the extent of misclassification and the factors associated with misclassification.

**What did the researchers do and find?:**

- We estimated that 1,166 law-enforcement-related deaths occurred in the US in 2015; The Counted captured a larger proportion of these deaths than the US mortality data.
- Law-enforcement-related deaths were most likely to be misclassified in mortality data if the death was not due to a gunshot wound or if it occurred in a low-income county.

**What do these findings mean?:**

- Datasets based on news media reports may offer higher-quality information on law-enforcement-related deaths than mortality data.
- Further exploration into the ways in which policymakers and public health officials report law-enforcement-related deaths is warranted.

## Introduction

The National Vital Statistics System (NVSS), administered by the US government and based on state death certificates, is the longest-running national data source on law-enforcement-related deaths (i.e., those involving fatal injuries inflicted by law enforcement), but has long been suspected of underreporting a large number of such deaths [1–3]. Other databases run by the US Department of Justice similarly undercount law-enforcement-related deaths [4]. In recent years, a new type of data source on legal intervention mortality has emerged: national databases maintained by newspapers, nongovernmental organizations, and the US Bureau of Justice Statistics (BJS; a governmental organization) that identify incidents via web searches of news media reports [3,5–8].

The NVSS has identified law-enforcement-related deaths since 1949, following the inclusion of “injury by intervention of police” as a diagnostic category in the 6th revision to the International Classification of Diseases (ICD) [9]. While the category has since been renamed as “legal intervention,” its definition remains unchanged up to the current ICD revision, ICD-10: “injuries inflicted by the police or other law-enforcing agents, including military on duty, in the course of arresting or attempting to arrest lawbreakers, suppressing disturbances, maintaining order, and other legal action” [10] (Table 1). A designation of legal intervention does not depend on whether the use of force resulting in the injury was lawful [11] or whether the injuries were inflicted intentionally.

**Table 1:** Definitions for law-enforcement-related deaths and reasons for underreporting in the National Vital Statistics System and The Counted.

Source	Term	Definition	Reasons for underreporting
National Vital Statistics System	“Legal intervention”	Based on the definition from the International Classification of Diseases–10th Revision (ICD-10): “injuries inflicted by the police or other law-enforcing agents, including military on duty, in the course of arresting or attempting to arrest lawbreakers, suppressing disturbances, maintaining order, and other legal action” [10]	Deaths will not appear if they are misclassified, i.e., assigned an ICD-10 code that does not correspond to legal intervention. This may happen because law enforcement involvement is not mentioned on the death certificate, or potentially due to coding errors by the National Center for Health Statistics.
The Counted	“People killed by police and other law enforcement agencies in the United States”	From The Counted website: “What is included in The Counted? Any deaths arising directly from encounters with law enforcement. This will inevitably include, but will likely not be limited to, people who were shot, tasered and struck by police vehicles as well those who died in police custody. What is not included in The Counted? Self-inflicted deaths during encounters with law enforcement. For instance, a person who died by crashing his or her vehicle into an oncoming car while fleeing from police at high speed is not regarded by the Guardian’s database to have been killed by law enforcement. The database does not include suicides or self-inflicted deaths including drug overdoses in police custody or detention facilities.” [12]	Deaths may not appear if they were unreported in news media, or if they were reported but The Counted staff did not identify these publications.

Prior studies found that NVSS counts of legal intervention deaths were lower in at least some US states compared to counts reported by law enforcement data sources, suggesting that the NVSS misses some proportion of these deaths [1,13,14]. This underreporting occurs when a death certificate is misclassified: it is wrongly assigned an ICD code that does not correspond to legal intervention, and the death can therefore not be identified as law-enforcement-related in queries of NVSS data (Table 1). Misclassification primarily occurs because the coroner or medical examiner certifying the death fails to mention police involvement in the literal text fields of the death certificate’s cause of death section (e.g., the field labeled “Describe how the injury occurred” does not state “killed by police”), although mistakes in the process of assigning ICD codes may still occur even when the death certificate indicates police involvement [15]. To our knowledge, there have been no prior national estimates of the misclassification rate for legal intervention deaths in the NVSS, nor has any research investigated factors associated with misclassification.

In recent years, a number of nongovernmental initiatives have sought to identify incidents of law-enforcement-related deaths in the US based on web searches of news media, and these databases provide counts that far exceed those reported in the NVSS and traditional US Department of Justice governmental data sources. Examples of such nongovernmental efforts include *The Guardian*’s The Counted (covering 2015–2016) [5], *The Washington Post*’s police shooting database (2015–present; excludes non-firearm deaths) [7], and Fatal Encounters (2014–present prospectively; 2000–2013 retrospectively) [6]. Prior analyses have found that, within the same time period, these sources report a nearly identical set of cases [16]. In addition to these nongovernmental efforts, the BJS redesigned its Arrest-Related Deaths (ARD) program in mid-2015 to track deaths in custody using a similar method: ARD first identifies cases based on a systematic internet search of news media reports, then requests more information about deaths from law enforcement agencies, medical examiners, and coroners [8]. Even as researchers have made increasing use of these news-media-based data sources [3,16–18] and the federal government has adopted their practices, there have been no prior estimates about the proportion of law-enforcement-related deaths that remain unreported in databases drawn from news media.

Our a priori hypotheses were that underreporting by the NVSS would exceed that by the news media sources, and that misclassification rates would be higher for decedents of color versus white, decedents in lower versus higher income counties, decedents killed by non-firearm versus firearm mechanisms, and deaths recorded by a medical examiner versus coroner. Our study aims to improve public health monitoring of law-enforcement-related deaths, which may ultimately aid efforts to improve accountability for both individual deaths and aggregate trends [18].

## Methods

We created a dataset of law-enforcement-related deaths in 2015 by matching 2 sources: The Counted, a news-media-based dataset created by the newspaper *The Guardian* [5], and the NVSS, from which we obtained individually identifiable mortality data for cases that were reported by *The Guardian*. Our study was deemed exempt from review by the Harvard T.H. Chan School of Public Health institutional review board (IRB16-1146) because it did not involve living persons. We were not able to publish death counts for all US states and counties due to privacy restrictions for NVSS data. We did not have a written prospective analysis plan; we agreed on an analytic plan at an October 2016 meeting and conducted all analyses in January 2017. Our cross-sectional study involved 2 main analyses: (1) a capture–recapture analysis to estimate the total number of law-enforcement-related deaths in the US during 2015, as well as the proportions captured by The Counted and the NVSS, and (2) a multilevel logistic regression analysis investigating the correlates of misclassification for law-enforcement-related deaths in NVSS data. This report has been prepared according to STROBE guidelines, as suggested by the Enhancing the QUAlity and Transparency Of health Research (EQUATOR) network (S1 STROBE checklist).

The Counted identified US law-enforcement-related deaths in 2015–2016 using web searches of news media reports; it defined these incidents as “any deaths arising directly from encounters with law enforcement … [such as] people who were shot, tasered and struck by police vehicles as well those who died in police custody” and excluded persons who died of self-inflicted injuries (Table 1) [12]. The website of the dataset also allowed members of the public to report cases; however, all deaths in the 2015–2016 dataset were substantiated based on local news media reports with the exception of 5 deaths identified via *The Guardian*’s original reporting [19]. *The Guardian* staff extracted characteristics of each incident including the decedent’s name, demographic information, street address of the police encounter, date of the injury occurrence, and mechanism of death. They also included a brief narrative description of events leading to the death. When necessary, reporting staff requested more information from local government agencies.

The NVSS receives electronic mortality data, based on death certificates, on deaths from all causes that are reported by 52 US-based independent registration areas (“states”; including the 50 states, District of Columbia, and New York City, which reports independently of New York State). On death certificates, funeral home directors record demographic information, and coroners or medical examiners report cause of death information. Staff at state vital statistics registries input death certificate information in a standardized electronic format. They send these data to the National Center for Health Statistics, which assigns up to 20 cause of death codes, following ICD-10, based on literal text written by the coroner/medical examiner. For a majority of decedents—approximately 60% of cases coded as legal intervention deaths in 2015—ICD codes are assigned by a computer program, SuperMICAR (National Center for Health Statistics; https://www.cdc.gov/nchs/nvss/mmds/super_micar.htm). Trained nosologists assign codes when automatic assignment fails.

### Exclusion criteria

The Counted used a broader definition for law-enforcement-related deaths than the NVSS, which follows the ICD definition for legal intervention (Table 1). Unlike the ICD definition, The Counted did not require that the injury be inflicted by a law enforcement officer and made no differentiation as to whether the injury was inflicted while a law enforcement officer was acting in the line of duty. To ensure that both datasets were comparable, we excluded cases from The Counted that did not conform to the ICD definition of legal intervention, while also recognizing that ambiguity in the ICD definition can make it unclear whether the diagnostic category is appropriate for certain instances. One category for which the definition lacks clarity is motor-vehicle-related deaths involving law enforcement. While on duty, an officer may accidently hit a pedestrian, although it is unclear whether this death occurred “in the course of arresting or attempting to arrest lawbreakers, suppressing disturbances, maintaining order, and other legal action.” Because these injuries may not specifically relate to the officer’s law enforcement role, we excluded decedents killed in motor-vehicle-related accidents unless they were being pursued by police or were intentionally injured in a police vehicle during transit. Another category for which definitional ambiguities arise is “deaths in custody,” i.e., non-firearm deaths that occur during the course of arrest or in holding cells and jails. In such instances, the circumstances of the death may be unknown to the public, and it may not be clear to death investigators whether actions by officers contributed to the death [20]. We excluded deaths in custody unless The Counted described a clear mechanism through which law enforcement actions may have caused the death (medical neglect, use of a chokehold, use of a Taser) or the death was reportedly ruled a homicide in The Counted’s narrative description (a homicide ruling can be made only if the injury was intentionally inflicted, while legal intervention, as defined by the ICD-10, does not require intentionality; however, a finding of homicide also provides evidence that law enforcement officers caused the death).

Additional exclusion criteria included instances of domestic violence perpetrated by law enforcement officers, as these did not occur in the course of carrying out “legal action.” For the same reason, we excluded deaths by “friendly fire” (i.e., an accidental shooting of one officer by another; the only such death reported in the 2015 The Counted data occurred during a training). Finally, we also excluded the small number of decedents (*N* = 3; <0.3% of deaths) who were injured in 2015 but died in 2016, as they would not appear in the 2015 mortality data.

### National Death Index plus matching process

The National Death Index (NDI) is a restricted-access database, administered by the National Center for Health Statistics, that researchers can use to access the same electronic mortality data reported in the NVSS [21,22]. Requestors submit a list of decedents, and the NDI returns either vital status only (i.e., confirmation of whether the individual has died) or, if the researcher pays a higher fee for “NDI Plus,” all reported ICD-10 coded causes of death for each decedent. For all cases meeting our inclusion criteria, we submitted names and years of birth (based on media-reported age) using NDI Plus. NDI Plus requires that submitted data include exact matches for first names, near matches for last names, and near matches for year of birth (±1 year) [22]. Matched records return the state in which the death occurred, date of death, and multiple ICD-10 coded causes of death. We identified true matches from NDI Plus output by ensuring dates and states of death were consistent with media reports. We considered the date of death to match when it fell within 4 days of the injury occurrence date reported in The Counted.

We rejected cases for which the date of death preceded the reported date of injury by more than 4 days. For cases whose NDI record reported a date of death more than 4 days after the reported injury, we flagged the result as a match only if we were able to locate a news article reporting the later date of death. Similarly, for deaths whose matched record reported a state that differed from the location of injury reported by The Counted, we flagged it as a match if we were able to locate a news article confirming the state of death (differing states for injury and death can happen if a person is transported across state lines to a hospital before the death). Finally, we tabulated the characteristics of matched cases and unmatched cases and stratified by measured covariates for comparison. Unmatched cases were not included in any subsequent analyses.

### Estimating the total number of law-enforcement-related deaths

Our first set of analyses used capture–recapture analysis (also known as multiple systems estimation) to estimate the number of US law-enforcement-related deaths in 2015. Using 2 or more matched, incomplete lists, capture–recapture analysis estimates the total size of a population, including the number of cases missed by all lists [23]. To conduct the capture–recapture analysis, we obtained monthly counts of deaths reported as legal intervention deaths in the 2015 NVSS public-use multiple cause of death file [24]. Using those counts along with the dataset derived from matching The Counted and NDI, we estimated the number of deaths (1) reported in The Counted only, (2) classified as legal intervention deaths in the NVSS only, and (3) reported in both systems. We considered a case to be reported as a legal intervention death in the NVSS when at least 1 of its multiple ICD-10 cause of death codes corresponded to legal intervention (ICD-10: Y35.0–Y35.4; Y35.6–Y35.7; Y89.0). We assumed unmatched cases from The Counted (95/1,086; 8.7%) were classified as legal intervention deaths in the NVSS at the same rate as matched cases: we added 43 of these deaths (45%) to the group that was captured by both the NVSS and The Counted, and added the remaining 52 cases (55%) to the group captured by The Counted only.

We used Poisson regression, with data stratified by 3-month periods, to conduct capture–recapture analysis. The counts for each group (deaths captured by The Counted only, the NVSS only, and both systems) analyzed by the Poisson model are presented in S1 Table. For capture–recapture analyses with only 2 data sources, the method assumes independence between the lists (i.e., the probability of a case appearing in one list is uncorrelated with its probability of appearing in the other list). This assumption is frequently violated in epidemiologic contexts, however: often there is positive list dependence, which leads to underestimated population sizes [25]. In our study, one possible source of list dependence is that both databases typically rely on reporting by police departments to ascertain cases, either when the agency issues press releases (in the case of media reports) or when it releases reports detailing the circumstances of the death to the coroner or medical examiner (in the case of the NVSS). With respect to the latter, journalists have revealed multiple incidents in which law enforcement agencies failed to release pertinent documents to death investigators for in-custody deaths or pressured death investigators to make a finding of non-homicide [26–28], although there is no evidence to suggest how frequently this occurs.

To address the potential for list dependence, we conducted a sensitivity analysis to estimate the maximum plausible number of cases adjusting for a prior correlation value between our 2 lists. We followed the method employed by Lum and Ball [29], who incorporated prior values, based on capture–recapture analyses of homicides from comparable sources in 5 other countries, when they estimated the number of law-enforcement-related deaths in the US from 2 probabilistically matched law enforcement datasets. By including the highest pairwise list correlation value they reported (0.93, based on a study of homicides in Syria) as an offset in our Poisson model, we calculated a maximum plausible estimate of the number of deaths in this sensitivity analysis.

### Analyzing correlates of misclassification in National Vital Statistics System mortality data

Our next set of analyses sought to identify correlates of misclassification of legal intervention deaths in NVSS mortality data, with misclassification defined as there not being any ICD-10 codes for legal intervention among the reported multiple causes of death. For the purpose of these analyses, we assumed The Counted’s matched cases were a random sample of the total population of US law-enforcement-related deaths in 2015. This is a tenable assumption because, as we report below, The Counted underreports relatively few incidents, and there appear to be no systematic differences between matched and unmatched cases. We used demographic data (age, gender, and race/ethnicity) reported in The Counted, which our prior research has found to be highly concordant with values reported on death certificates [15]. We also used The Counted data on mechanism of death (firearm or non-firearm) and the county where the fatal injury occurred. At the county level, we identified median household income quintiles based on 2011–2015 US Census data [30], urbanicity based on National Center for Health Statistics classifications [31], and death investigator type (medical examiner, elected coroner, or appointed coroner) based on a Centers for Disease Control and Prevention (CDC) dataset [32]. For counties with ambiguous CDC data regarding death investigator type, we contacted local government agencies directly.

After tabulating descriptive statistics on misclassified and properly classified legal intervention deaths, we calculated and mapped misclassification rates by state. We then conducted multilevel logistic regression, using Stata version 14.2 (StataCorp [https://www.stata.com]), to model the odds of misclassification. Our univariable and multivariable models included random intercepts for counties and states. We used post-estimation commands to calculate the average marginal effects for select covariates, and we report these as predicted probabilities of misclassification.

## Results

The Counted identified 1,146 law-enforcement-related deaths in the US during 2015. Applying our exclusion criteria, we eliminated 60 cases that did not conform to the ICD definition of legal intervention, such that the initial dataset included 1,086 observed deaths (Table 2).

**Table 2:** Cases included and excluded as legal intervention deaths from The Guardian’s The Counted database of law-enforcement-related deaths (US, 2015).

Category	Number
**Total cases reported**	1,146
**Exclusion criteria**	
Struck by vehicle, unless decedent was injured by law enforcement vehicle during pursuit or was intentionally injured as a passenger during transport	27
Domestic violence	6
In-custody death, unless it followed use of a Taser/chokehold, involved withholding essential care (e.g., medical care or water), or was reported by The Counted as having been ruled a homicide by the coroner/medical examiner	23
Injury occurred in 2015, but death occurred in 2016	3
“Friendly fire” (officer accidently shot by another officer)	1
**Total cases excluded**	60
**Total cases included as 2015 legal intervention deaths**	1,086

Among the 1,086 observed cases, the majority were ages 18–44 years (766/1,086; 70.5%), were men (1,043/1,086; 90.6%), were killed by a firearm (1,008/1,086; 92.8%), resided in a large metro area (583/1,086; 53.7%), and had their death reported by a medical examiner (682/1,086; 57.8%) (Table 3). Additionally, 27.1% (294/1,086) of decedents were black, 17.2% were Hispanic (187/1,086), 1.1% were American Indian (12/1,086), 2.0% were Asian/Pacific Islander (22/1,086), 50.9% were white non-Hispanic (553/1,086), and 1.7% were of unknown race/ethnicity (18/1,086) (Table 3); the corresponding national estimates for the racial/ethnic composition of the US population in 2015 were 13.0% black, 17.6% Hispanic, 0.8% American Indian, 5.9% Asian/Pacific Islander, and 62.6% white non-Hispanic [33].

**Table 3:** Characteristics of law-enforcement-related deaths from The Counted matched and unmatched to National Vital Statistics System mortality records using the National Death Index (US, 2015).

Characteristic	Matched cases	Unmatched cases	Total cases	Percent matched (95% CI)	*p-*Value (Fisher’s exact test)^1^
***Total sample***	991	95	1,086	91.3% (89.4, 92.9)	n/a
***Individual-level characteristics***					
**Age**					0.61
Less than 18 years	16	1	17	94.1% (71.3, 99.9)	
18 to 44 years	704	62	766	91.9% (89.4, 93.7)	
45 years and older	271	30	301	90.0% (86.1, 93.2)	
Missing	0	2	2	0.0% (0.0, 84.2)	
**Gender**					0.09
Men	955	88	1,043	91.6% (89.7, 93.2)	
Women	36	7	43	83.7% (69.3, 93.2)	
Missing	0	0	0	n/a	
**Race/ethnicity**^2^					0.12
Black	265	29	294	90.1% (86.1, 93.2)	
White	516	37	553	93.3% (90.1, 95.2)	
Hispanic	164	23	187	87.7% (82.1, 92.0)	
American Indian/Alaska Native	11	1	12	91.7% (61.5, 99.8)	
Asian/Pacific Islander	21	1	22	95.5% (77.2, 99.9)	
Missing	14	4	18	77.8% (52.4, 93.6)	
**Mechanism of death**					0.84
Firearm	920	88	1,008	91.3% (89.4, 92.9)	
Non-firearm	71	7	78	91.0% (82.4, 96.3)	
Missing	0	0	0	n/a	
***County-level characteristics***					
**Death investigator type**					0.05
Medical examiner	582	46	628	92.7% (90.4, 94.6)	
Coroner (elected)	380	43	423	89.8% (86.6, 92.5)	
Coroner (appointed)	29	6	35	82.9% (66.4, 93.4)	
Missing	0	0	0	n/a	
**Urbanicity**					0.20
Large metro–central	358	33	391	91.6% (88.4, 94.1)	
Large metro–fringe	171	21	192	89.1% (83.8, 93.1)	
Medium metro	223	14	237	94.1% (90.3, 96.7)	
Small metro	70	12	82	85.4% (75.8, 92.2)	
Micropolitan	71	6	77	92.2% (83.8, 97.1)	
Non-core	98	8	106	92.5% (85.7, 96.7)	
Missing	0	0	0	n/a	
**County median income (quintiles)**					0.19
Q5 (highest income)	198	18	216	91.7% (87.1, 95.0)	
Q4	189	20	209	90.4% (85.6, 94.1)	
Q3	201	23	224	89.7% (85.0, 93.4)	
Q2	196	10	206	95.1% (91.3, 97.6)	
Q1 (lowest income)	207	24	231	89.6% (84.9, 93.2)	
Missing	0	0	0	n/a	

^1^p-Values are for a difference in matching rate across categories of a characteristic; p-values are not adjusted for clustering and are therefore biased downward for county-level variables.

^2^Other than Hispanic, all races are non-Hispanic.

n/a, not applicable.

We identified true matches for 91.3% (991/1,086) of included cases using NDI Plus (Table 3). Matching rates were lower than 85% for decedents who were women (36/43; 83.7%), had missing race/ethnicity (14/18; 77.8%), or had death certified by an appointed coroner (29/35; 82.9%). Results from Fisher’s exact tests show that among these individual- and county-level characteristics, the only variable for which differences in matching rates were statistically significant (*p* = 0.049) was the death investigator type. These tests did not adjust for clustering by counties, however; *p*-values for county-level variables are therefore biased downward and may suggest statistically significant differences in matching rates where none exist.

Among the 991 matched cases, firearm deaths comprised 92.8%, or 920/991 cases (Table 4). The second most common mechanism was death due to Taser (46/991; 4.6%). This was followed by struck by/against injuries (18/991; 1.8%), motor-vehicle-related injuries (5/991; 0.4%), and neglect (3/991; 0.3%).

**Table 4:** National Vital Statistics System cause of death codes, by mechanism of death, for law-enforcement-related deaths matched to The Counted.

Mechanism of death (percent of total; 95% CI)	Underlying cause of death as reported in the National Vital Statistics System (ICD-10 range)^1^																	
	All		Legal intervention (Y35; Y89.0)^2^		Assault (X95–Y09)		Events of undetermined intent or cause missing (Y10–Y34; R99)^3^		Suicide (X60–X84)		Accident (V01–X59)		Circulatory/respiratory diseases (I00–J99)		Mental/behavioral disorders (F00–F99)		Other causes of death	
	*N*	Percent	*N*	Percent (95% CI)	*N*	Percent (95% CI)	*N*	Percent (95% CI)	*N*	Percent (95% CI)	*N*	Percent (95% CI)	*N*	Percent (95% CI)	*N*	Percent (95% CI)	*N*	Percent (95% CI)
All (100.0%)	991	100.0	444	44.8 (41.7, 48.0)	471	47.5 (44.4, 50.7)	22	2.2 (1.4, 3.3)	16	1.6 (0.9, 2.6)	15	1.5 (0.8, 2.5)	14	1.4 (0.8, 2.4)	7	0.7 (0.3, 1.4)	2	0.2 (0.0, 0.7)
Firearm (92.8%; 91.0, 94.4)	920	100.0	434	47.2 (43.9, 50.4)	456	49.6 (46.4, 52.8)	11	1.2 (0.6, 2.1)	16	1.7 (1.0, 2.8)	1	0.1 (0.0, 0.6)	2	0.2 (0.0, 0.7)	0	0.0 (0.0, 0.4)	0	0.0 (0.0, 0.4)
Taser (4.6%; 3.4, 6.1)	46	100.0	6	13.0 (4.9, 26.3)	10	21.7 (10.9, 36.4)	8	17.4 (7.8, 31.4)	0	0.0 (0.0, 7.7)	10	21.7 (10.9, 36.4)	7	15.2 (6.3, 28.9)	5	10.9 (3.6, 23.6)	0	0.0 (0.0, 7.7)
Struck by/against (1.8%; 1.1, 2.9)	18	100.0	4	22.2 (6.4, 47.6)	4	22.2 (6.4, 47.6)	2	11.1 (1.4, 34.7)	0	0.0 (0.0, 18.5)	1	5.6 (0.1, 27.3)	5	27.8 (9.7, 53.5)	2	11.1 (1.4, 34.7)	0	0.0 (0.0, 18.5)
Motor vehicle (0.4%; 0.1, 1.0)	4	100.0	0	0.0 (0.0, 60.2)	1	25.0 (0.6, 80.6)	0	0.0 (0.0, 60.2)	0	0.0 (0.0, 60.2)	3	75.0 (19.4, 99.4)	0	0.0 (0.0, 60.2)	0	0.0 (0.0, 60.2)	0	0.0 (0.0, 60.2)
Neglect (0.3%; 0.1, 0.9)	3	100.0	0	0.0 (0.0, 70.6)	0	0.0 (0.0, 70.6)	1	33.3 (0.8, 90.6)	0	0.0 (0.0, 70.6)	0	0.0 (0.0, 70.6)	0	0.0 (0.0, 70.6)	0	0.0 (0.0, 70.6)	2	66.7 (0.9, 99.2)

^1^Mortality records report 1 underlying cause of death, defined as “(a) the disease or injury which initiated the train of events leading directly to death, or (b) the circumstances of the accident or violence which produced the fatal injury” [22]. The records also report up to 20 “multiple causes of death” based on any other health conditions reported on the death certificate. In rare instances (N = 2), legal intervention was reported as a multiple cause of death but not an underlying cause of death. We nonetheless present these cases in the column for legal intervention.

^2^Excludes legal execution, Y35.5.

^3^A classification of “events of undetermined intent” signifies that the coder knew (based on death certificate literal text) that the cause of death involved external injuries, but could not identify whether the injury was due to legal intervention, assault, suicide, or accident. “Missing” signifies that the coder was unable to make any determination whatsoever about cause of death.

ICD-10, International Classification of Diseases–10th Revision.

Overall, 444 (44.8%) of the law-enforcement-related deaths were properly classified as legal intervention deaths in the NVSS. The most common underlying cause of death for misclassified cases was assault, which was more prevalent than legal intervention and accounted for 47.5% of all matched cases (*N* = 471). While nearly all firearm deaths were coded as legal intervention or assault (96.8% combined), the causes of death reported for non-firearm mechanisms were more heterogeneous. Deaths that followed the use of Tasers were reported as legal intervention (6/46; 13%), assault (10/46; 21.7%), missing/undetermined (8/46; 17.4%), accidental injury (10/46, 21.7%), and mental health/behavioral disorders (5/46; 10.9%). Struck by/against was the only other non-firearm mechanism for which any cases were classified as legal intervention (4/18 struck by/against injuries; 22.2%).

### Estimates of the number of US law-enforcement-related deaths in 2015

**Fig 1:**
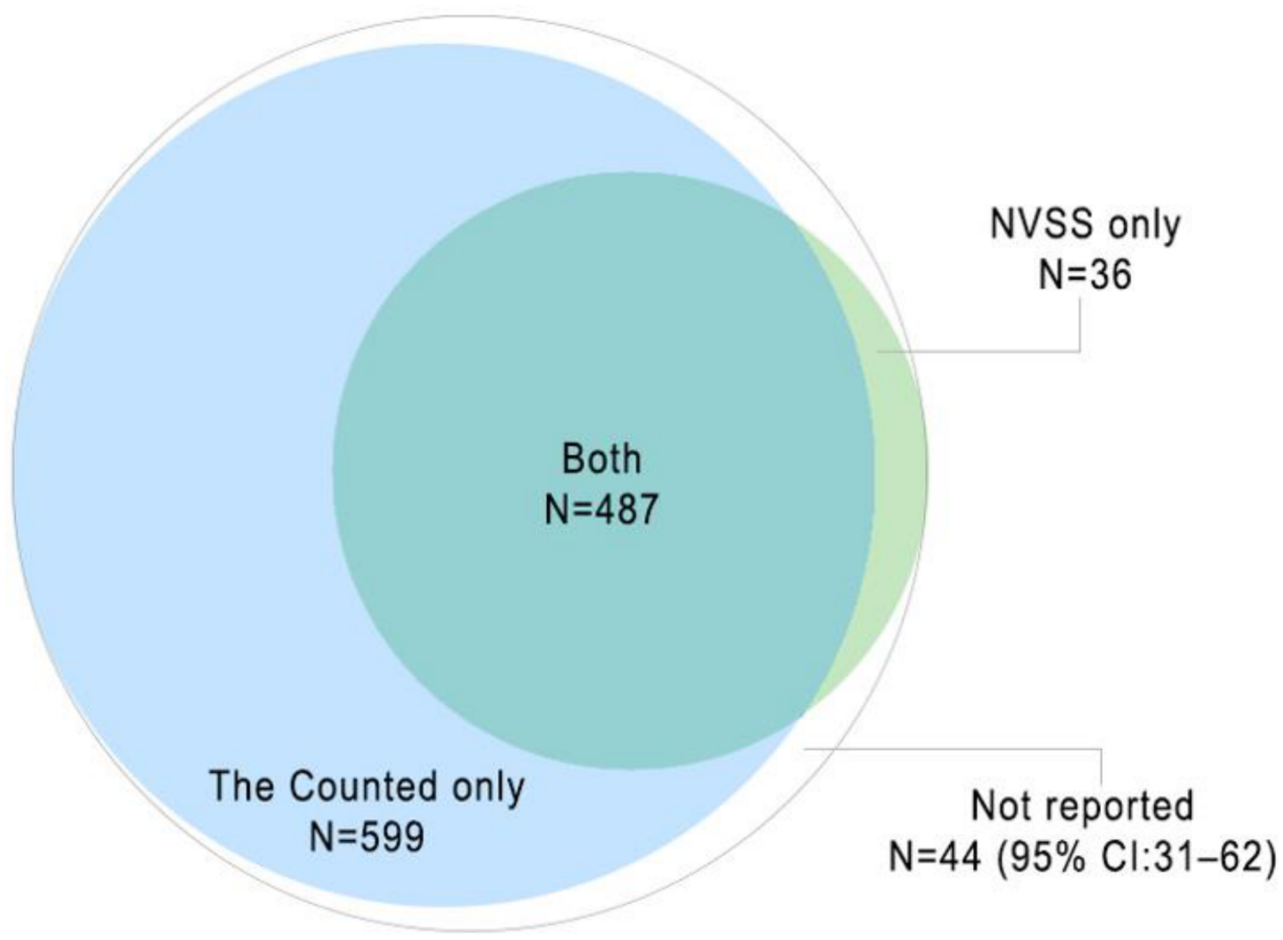
Two-source estimate, assuming independence between lists, of the total number of law-enforcement-related deaths in the US, 2015 (N = 1,166; 95% CI: 1,153, 1,184). NVSS, National Vital Statistics System.

There were 599 deaths reported in The Counted only, 36 reported in the NVSS only, 487 reported in both lists, and an estimated 44 (95% CI: 31, 62) not reported in either list. Assuming independence between lists, our capture–recapture model estimates that the total number of US law-enforcement-related deaths in 2015 was 1,166 (95% CI: 1,153, 1,184) (Fig 1). This suggests that the NVSS documented 44.9% (95% CI: 44.2%, 45.4%) of law-enforcement-related deaths, and The Counted documented 93.1% (95% CI: 91.7%, 94.2%). Our sensitivity analyses show that these estimates are robust to potential pairwise list correlation. Assuming the highest of the pairwise list correlation values reported by Lum and Ball [29], 0.93, the maximum number of deaths was only slightly higher, equaling 1,233 (95% CI: 1,200, 1,280). Under this maximum scenario, The Counted documented 88.1% (95% CI: 84.8%, 90.5%) of cases, and the NVSS documented 42.4% (95% CI: 40.9%, 43.6%).

### Correlates of ICD-10 misclassification of law-enforcement-related deaths

**Fig 2:**
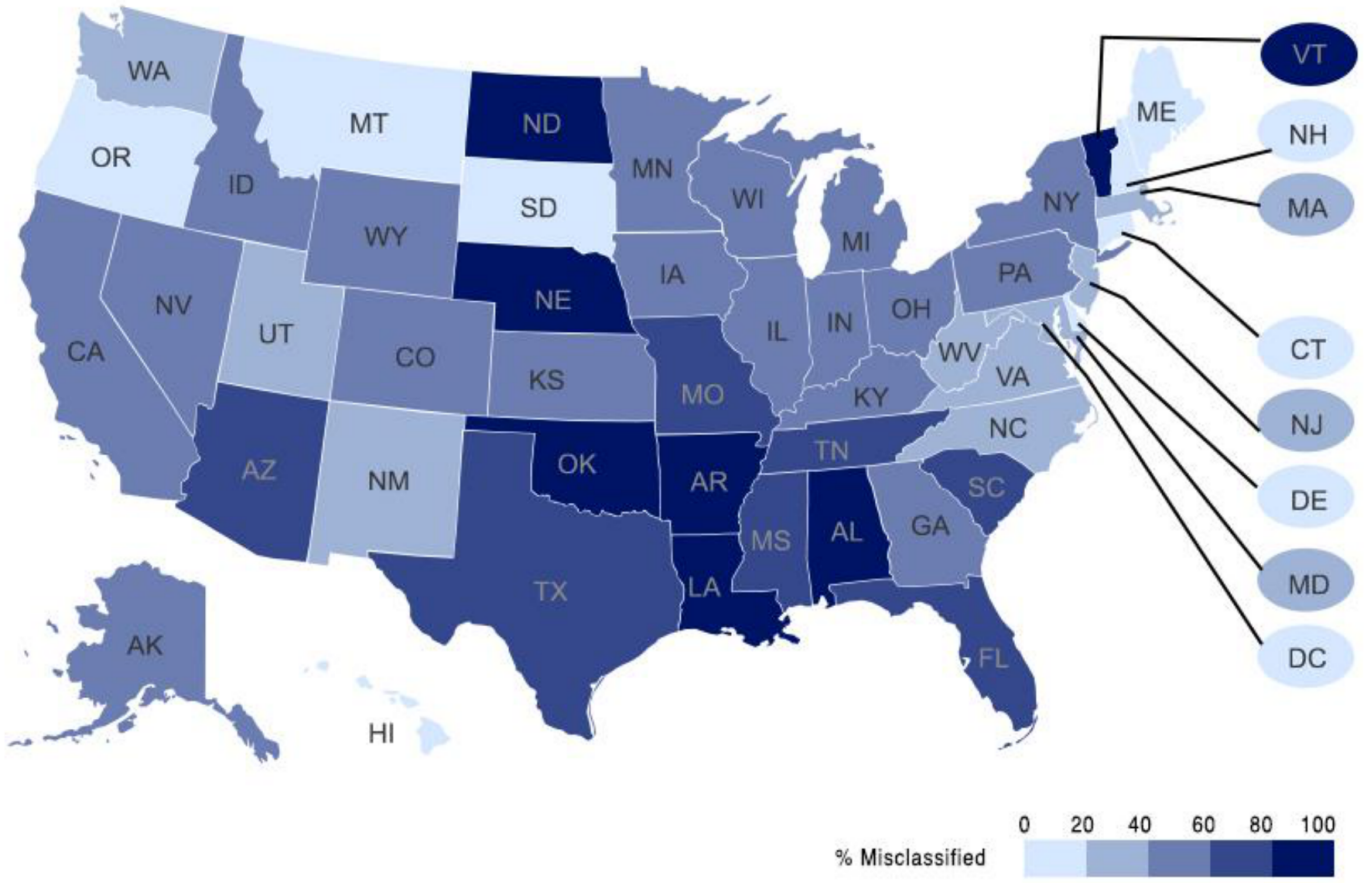
Law-enforcement-related death misclassification rates by state (2015; N = 991). Rhode Island is not displayed because there were zero matched cases from the state in the dataset. This map is based on an image by Paul Robinson, available at https://commons.wikimedia.org/wiki/File:Labelled_US_map.svg.

We found that, among cases reported in The Counted and matched to NVSS data, 55.2% (547/991) were misclassified in the NVSS. These deaths occurred in 51 states (49 states, the District of Columbia, and New York City; The Counted did not report any cases from Rhode Island meeting our inclusion criteria) (Table 5; Fig 2) and in 491 of 3,144 US counties. Misclassification rates ranged from 0% to 100%; among states with ≥10 matched cases, rates ranged from 17.6% (Washington) to 100.0% (Oklahoma). Taken together, 5 states—California, Texas, Florida, Oklahoma, and Arizona—contained 42.4% of matched cases and accounted for a majority of the misclassified cases (50.3%). Among these 5 states, misclassification was 40% to <60% in 1 state (California), 60% to <80% in 3 states (Arizona, Florida, and Texas), and ≥80% in 1 state (Oklahoma).

**Table 5:** Misclassification rates for law-enforcement-related deaths in National Vital Statistics System mortality data based on cases matched to The Counted, 2015 (N = 991).

Percent misclassified	State (abbreviation) by number of deaths		
	<10 deaths	10 to <20 deaths	≥20 deaths
<20%	Connecticut (CT), Delaware (DE), District of Columbia (DC), Hawaii (HI), Maine (ME), Montana (MT), New Hampshire (NH), South Dakota (SD)	Oregon (OR)	(None)
20 to <40%	West Virginia (WV)	Maryland (MD), Massachusetts (MA), New Jersey (NJ), New Mexico (NM), Utah (UT), Virginia (VA)	North Carolina (NC), Washington (WA)
40 to <60%	Idaho (ID), New York City^1^, Wyoming (WY)	Kansas (KS), Kentucky (KY), Michigan (MI), Minnesota (MN), Nevada (NV), New York (NY), Wisconsin (WI)	California (CA), Colorado (CO), Georgia (GA), Illinois (IL), Indiana (IN), Ohio (OH), Pennsylvania (PA)
60 to <80%	Alaska (AK), Iowa (IA)	Mississippi (MS), Missouri (MO), South Carolina (SC), Tennessee (TN)	Arizona (AZ), Florida (FL), Texas (TX)
≥80%	Arkansas (AR), North Dakota (ND), Nebraska (NE), Vermont (VT)	Alabama (AL)	Louisiana (LA), Oklahoma (OK)

The matched dataset did not include any deaths from Rhode Island.

^1^New York City reports deaths to the National Vital Statistics System independently of New York State.

In descriptive tabulations (Table 6), groups for whom misclassification rates exceeded 60% included decedents age <18 years (11/16; 68.8%), black decedents (162/265; 61.1%), decedents with a non-firearm mechanism of death (61/71; 85.9%), and those who died in a county in the second lowest income quintile (124/196; 63.3%). Misclassification rates were lower than 40% among persons who were Asian/Pacific Islander (8/21; 38.1%) and those who died in the highest income counties (76/198; 38.4%). Chi-squared tests of independence found that misclassification rates exhibited statistically significant differences by race/ethnicity (*p* = 0.04), mechanism of death (*p* < 0.01), and county income quintile (*p* < 0.01).

**Table 6:** Characteristics of misclassified and properly classified law-enforcement-related deaths in the National Vital Statistics System, based on incidents identified in The Counted (US, 2015).

Characteristic	Misclassified cases	Properly classified cases	Total cases	Percent misclassified (95% CI)	*p-*Value (χ^2^ test of independence)^1^
***Total sample***	547	444	991	55.2%	n/a
***Individual-level characteristics***					
**Age**					
Less than 18 years	11	5	16	68.8% (41.3, 89.0)	0.27
18 to 44 years	395	306	701	56.3% (52.6, 60.1)	
45 years and older	141	130	271	52.0% (45.9, 58.1)	
Missing	0	0	0	n/a	
**Gender**					
Men	526	429	955	55.1% (51.9, 58.3)	0.70
Women	21	15	36	58.3% (40.8, 74.9)	
Missing	0	0	0	n/a	
**Race/ethnicity**^2^					
Black	162	103	265	61.1% (55.0, 67.0)	0.04
White	266	250	516	51.6% (47.1, 55.9)	
Hispanic	97	67	164	59.1% (51.2, 66.7)	
American Indian/Alaska Native	6	5	11	54.5% (23.4, 83.3)	
Asian/Pacific Islander	8	13	21	38.1% (18.1, 61.2)	
Missing	8	6	14	57.1% (28.9, 82.3)	
**Mechanism of death**					
Firearm	486	434	920	52.8% (49.5, 56.1)	<0.01
Non-firearm	61	10	71	85.9% (75.6, 93.0)	
Missing	0	0	0	n/a	
***County-level characteristics***					
**Death investigator type**					
Medical examiner	320	262	582	55.0% (50.8, 59.1)	0.90
Coroner (elected)	212	168	380	55.8% (50.6, 60.9)	
Coroner (appointed)	15	14	29	51.7% (32.5, 70.6)	
Missing	0	0	0	n/a	
**Urbanization**					
Large metro–central	210	148	358	58.7% (53.4, 63.8)	0.32
Large metro–fringe	85	86	171	49.7% (42.0, 57.4)	
Medium metro	129	94	223	57.8% (51.1, 64.4)	
Small metro	35	35	70	50.0% (37.8, 62.2)	
Micropolitan	37	34	71	52.1% (40.0, 64.1)	
Non-core	51	47	98	52.0% (41.2, 62.2)	
Missing	0	0	0	n/a	
**County median income (quintiles)**					
Q5 (highest income)	76	122	198	38.4% (31.6, 45.5)	<0.01
Q4	111	78	189	58.7% (51.4, 65.8)	
Q3	116	85	201	57.7% (50.6, 64.6)	
Q2	124	72	196	63.3% (56.1, 70.0)	
Q1 (lowest income)	120	87	207	58.0% (50.9, 64.8)	
Missing	0	0	0	n/a	

^1^p-Values are not adjusted for clustering and are therefore biased downward for county-level variables.

^2^Other than Hispanic, all races are non-Hispanic.

n/a, not applicable.

The multivariable mixed-effects logistic model (Table 7), which controlled for all individual- and county-level covariates, identified statistically significant differences in misclassification rates by mechanism of death (odds ratio [OR] for non-firearm versus firearm: 68.2; 95% CI: 15.7, 297.5; *p* < 0.01) and county median household income quintile (OR: 10.1; 95% CI: 2.4, 42.8; *p* < 0.01). Using average values for all other covariates, the predicted probability of misclassification for firearm deaths was 48.6% (95% CI: 41.5%, 55.6%), while for non-firearm deaths it was 86.4% (95% CI: 78.2%, 94.6%). For deaths occurring in the highest-income-quintile counties, the predicted probability of misclassification was 33.4% (95% CI: 23.3%, 43.5%), while among the lowest-income-quintile counties the probability was 57.2% (95% CI: 46.8%, 67.6%). Finally, there was 2.7 times more variability in misclassification rates within states (county-level variance for random intercepts = 7.1) than between states (variance = 2.7).

**Table 7:** Multilevel logistic regression models for the relative odds of misclassification of law-enforcement-related deaths in National Vital Statistics System mortality data (US, 2015; N = 991).

Characteristic	Univariable models			Multivariable model (controlling for all variables below)		
	OR	95% CI	*p-*Value	OR	95% CI	*p-*Value
***Individual-level characteristics***						
**Age**						
Less than 18 years	2.14	0.33, 13.94	0.42	2.34	0.34, 15.98	0.39
18 to 44 years (referent)	1.00	—	—	1.00	—	—
45 years and older	0.96	0.59, 1.58	0.88	1.01	0.58, 1.76	0.98
**Gender**						
Men (referent)	1.00	—	—	1.00	—	—
Women	1.24	0.44, 3.53	0.69	1.46	0.48, 4.46	0.51
**Race/ethnicity**^1^						
Black	1.50	0.85, 2.66	0.16	1.27	0.67, 2.42	0.46
White (referent)	1.00	—	—	1.00	—	—
Hispanic	1.44	0.75, 2.77	0.27	1.33	0.66, 2.68	0.42
American Indian/Alaska Native	1.70	0.16, 17.81	0.66	1.31	0.12, 13.91	0.83
Asian/Pacific Islander	0.67	1.59, 2.84	0.59	0.71	0.15, 3.30	0.66
**Mechanism of death**						
Firearm (referent)	1.00	—	—	1.00	—	—
Non-firearm	63.74	15.11, 268.77	<0.01	68.24	15.65, 297.46	<0.01
***County-level characteristics***						
**Death investigator type**						
Medical examiner (referent)	1.00	—	—	1.00	—	—
Coroner (elected)	1.57	0.65, 3.78	0.31	1.85	0.66, 5.18	0.24
Coroner (appointed)	1.23	0.10, 14.87	0.87	1.01	0.07, 15.59	0.99
**Urbanization**						
Large metro–central	1.22	0.44, 3.38	0.70	1.53	0.48, 4.89	0.48
Large metro–fringe	1.41	0.50, 3.97	0.51	4.00	1.14, 14.03	0.03
Medium metro (referent)	1.00	—	—	1.00	—	—
Small metro	0.92	0.31, 2.76	0.89	1.03	0.31, 3.38	0.96
Micropolitan	0.58	0.17, 1.97	0.38	0.35	0.09, 1.40	0.14
Non-core	0.62	0.18, 2.09	0.44	0.53	0.13, 2.08	0.36
**County median income (quintiles)**						
Q5 (highest income; referent)	1.00	—	—	1.00	—	—
Q4	3.58	1.09, 11.78	0.04	8.32	2.00, 34.57	<0.01
Q3	3.46	1.08, 11.05	0.04	7.02	1.75, 28.17	<0.01
Q2	3.54	1.16, 10.89	0.03	10.39	2.52, 42.82	<0.01
Q1 (lowest income)	2.71	0.92, 7.97	0.07	10.11	2.39, 42.82	<0.01
**Variance: county random intercepts | state**	—			7.12		
**Variance: state random intercepts**	—			2.67		

^1^Other than Hispanic, all races are non-Hispanic.

OR, odds ratio.

## Discussion

We estimated the total number of law-enforcement-related deaths in the US in 2015–1,166 deaths (95% CI: 1,153, 1,184)—and found that, as hypothesized, a much higher proportion of such deaths were captured by *The Guardian*’s The Counted (93.1%; 95% CI: 91.7%, 94.2%) than by US vital statistics data (44.9%; 95% CI: 44.2%, 45.4%). We also found that misclassification rates in NVSS data for law-enforcement-related deaths varied widely both within and between states, and that misclassification was more likely for non-firearm deaths than firearm deaths and for deaths that occurred outside of the highest income counties. These findings together affirm that major shortcomings exist in official counts of law-enforcement-related deaths based on US vital statistics. The results additionally suggest these shortcomings could potentially be corrected by simultaneously (1) improving the extent and accuracy of the information recorded in death certificates and (2) expanding the types of data employed (such as media-based reports) utilized to generate official counts of these cases.

Our study is strengthened by its use of identifiable, national US mortality data to estimate the number of law-enforcement-related deaths and to analyze patterns of misclassification of these deaths in the NVSS. One limitation is that differential matching rates for our NVSS/The Counted dataset may bias results, although the high proportion of cases that we were able to match limits this bias. Additionally, we were unable to examine the characteristics of cases that were unreported in The Counted. One issue of concern is that law-enforcement-related deaths occurring in rural areas may not be reported in the news media, because there is less local news coverage available in rural areas and rural news sources may not be accessible on the internet [34]. Another issue is that we cannot know with complete certainty in which county the death was declared; The Counted reports the location where the fatal injury was inflicted. While data from California suggest that four-fifths of persons fatally injured by law enforcement die immediately [35], an unknown proportion of the remaining one-fifth may die at a hospital in another county. Facilities best equipped to treat gunshot wounds, such as level I trauma centers, are more likely to be located in urban and higher income counties [36], so this could lead to measurement error for county-level variables. Finally, The Counted data do not include deaths that occurred in 2015 due to an injury inflicted in 2014, so any such cases are absent from the analyses. However, this is likely a very small number of cases (for injuries inflicted in 2015, we identified only 3 cases, or <0.3% of deaths, for which the death occurred in 2016).

Our estimates, derived from capture–recapture analysis, for the total number of law-enforcement-related deaths in 2015 are robust to pairwise list dependence. Because of the high degree of overlap between our 2 data sources (i.e., a large proportion of deaths reported in the NVSS were also reported in The Counted), any potential list dependency had minimal effect on the overall estimate. The Counted was more effective at identifying deaths: a case was approximately twice as likely to be reported in The Counted compared to the NVSS. Comparing its coverage rate to previous estimates produced by the BJS, The Counted outperformed ARD (which captured an estimated 49% of deaths over the period 2003–2011, excluding 2010) as well as the FBI’s Supplementary Homicide Reports data (which captured an estimated 46% of deaths over the same period) [4].

Only 2 prior studies have used capture–recapture analysis to estimate the number of US law-enforcement-related deaths. First, a BJS analysis for the period 2003–2011 (excluding 2010) was based on probabilistically matched deaths from 2 national law enforcement sources and estimated that there were on average 928 annual law-enforcement-related deaths in the US [4]. The authors of the BJS study note that many law enforcement agencies did not report any deaths to either system, and, once they accounted for nonresponse, their estimate was approximately 1,200, on par with our estimate. Second, Lum and Ball [29], adjusting for potential list dependency but not for agency nonresponse, used the same BJS data to estimate an annual mean of 1,500 deaths in the US, which is higher than our estimate. They state that adjusting for nonresponse would increase their estimate by an additional 30%. Differences between these prior estimates and our own may be attributable to (1) an actual change in the incidence of law-enforcement-related deaths, (2) uncertainty in the magnitude of list dependence, or (3) potential error in the prior estimates introduced by the imprecision of probabilistic matching.

We found that the majority of misclassified cases for the most common cause of death—fatal gunshot wounds by law enforcement—were incorrectly coded as assault. As hypothesized, a higher risk of misclassification occurred for the less common phenomenon of law-enforcement-related deaths involving injury mechanisms other than firearms. This may reflect a lack of consensus among coroners and medical examiners about how to report non-firearm deaths in police custody [37]. Notably, cause of death classification was especially inaccurate for law-enforcement-related deaths due to Taser shocks, which was the second most common mechanism after firearms.

While misclassification of law-enforcement-related deaths is a problem throughout the country, affecting 55% of mortality records nationally, the probability of misclassification varied widely both within and between states, and also by social and economic groups. Descriptive analyses found higher probabilities of misclassification among decedents who were under age 18 years, black, or residing in the poorest county income quintiles, suggesting researchers should exercise caution when comparing rates of law-enforcement-related mortality among various sociodemographic groups using only national-level data. However, in our analyses that accounted for systematic differences in odds of misclassification by state and county (i.e., the multilevel models), only county income quintile remained significantly associated with risk of misclassification. Possible explanations for the inverse association between county income and odds of misclassification may include better resources and training among coroners/medical examiners in wealthier counties and differences in the political culture in wealthier counties that lead to greater transparency in relation to law-enforcement-related deaths. Even so, contrary to our hypotheses, we did not find that misclassification differed by death investigator type. It may be that extent of training and resources matters more to mitigate misclassification than death investigator type.

Misclassification of cause of death is a longstanding and ongoing concern in US vital statistics, and the validity of these reported data may vary widely depending on the type of disease or injury [38,39]. However, evidence suggests that the accuracy of mortality classification for homicide—an outcome similar to law-enforcement-related mortality in that it is also certified by coroners and medical examiners—is very high. A prior study of large US cities found a near-perfect correlation between homicide counts reported in the NVSS and homicide counts reported in Supplementary Homicide Reports [40]. For law-enforcement-related deaths, however, correlations between the same 2 systems are considerably lower [1,13].

### Future research and implications

Future studies could estimate the number of law-enforcement-related deaths, nationally or subnationally, using data from additional years and sources. Alternative data sources for these deaths include the National Violent Death Reporting System (NVDRS), which covers 40 US states and the District of Columbia as of 2017 [41], and deaths-in-custody lists maintained by the attorneys general of California [35] and Texas [42]. Additionally, state offices of vital statistics and departments of health can identify the shortcomings of their current vital statistics data by reviewing death certificates for law-enforcement-related deaths. It will also be useful to evaluate whether making such deaths a notifiable condition improves reporting [9], per new legislation enacted in Tennessee in 2017 [43].

There are multiple interventions that may improve public health monitoring of law-enforcement-related deaths. Examples include training medical examiners and coroners to indicate law enforcement involvement in death certificate literal text, increasing the use of news media reports as a data source for NVDRS states, and legally requiring disclosure of these deaths to health departments [43] or death investigators [27]. Additionally, health departments can create websites to provide the public with real-time reports of law-enforcement-related deaths that occur within their jurisdiction. This can be coupled with the inclusion of such deaths in a jurisdiction’s list of notifiable conditions, which would allow for reporting of these deaths to health departments by medical staff, first responders, and members of the public [18].

Improving public health monitoring of law-enforcement-related mortality is a critical part of efforts to ensure public accountability for these incidents and prevent future incidents. Also warranting attention is improved monitoring of nonfatal injuries due to law enforcement, which currently are not captured by any official or media-based reporting system [44]. Better-quality data would allow researchers to quantify various forms of social inequality that may be linked to law-enforcement-related mortality (e.g., differences by race/ethnicity, socioeconomic position, and gender identity), compare rates between jurisdictions, and identify whether incidence is increasing or decreasing over time [18,44].

## Supporting information

S1 STROBE Checklist (PDF)

S1 Table Counts from the National Vital Statistics System, the National Death Index, and The Counted for capture–recapture. (XLSX)

## Abbreviations

ARD: Arrest-Related Deaths
BJS: US Bureau of Justice Statistics
ICD: International Classification of Diseases
ICD-10: International Classification of Diseases–10th Revision
NDI: National Death Index
NVDRS: National Violent Death Reporting System
NVSS: National Vital Statistics System
OR: odds ratio

## References

[1] Loftin C, McDowall D, Xie M. Underreporting of homicides by police in the United States, 1976–2013. Homicide Stud. 2017;21:159–74. doi: 10.1177/1088767917693358

[2] Sherman LW, Langworthy RH. Measuring homicide by police officers. J Crim Law Criminol. 1979;70:546–60.

[3] Zimring FE. When police kill. Cambridge (Massachusetts): Harvard University Press; 2017.

[4] Banks D, Couzens L, Blanton C, Cribb D. Arrest-Related Deaths program assessment. Washington (DC): Bureau of Justice Statistics; 2015 [cited 2017 Sep 1]. http://www.bjs.gov/content/pub/pdf/ardpatr.pdf.

[5] The Counted: people killed by police in the US—database. The Guardian; 2016 [cited 2016 Nov 1]. http://www.theguardian.com/thecounted.

[6] Fatal Encounters. 2016 [cited 2016 Nov 1]. http://fatalencounters.org.

[7] 995 people shot dead by police in 2015. Washington (DC): Washington Post; 2016 [cited 2016 Nov 1]. https://www.washingtonpost.com/graphics/national/police-shootings/.

[8] Banks D, Ruddle P, Kennedy E, Planty MG. Arrest-Related Deaths program redesign study, 2015–16: preliminary findings. Washington (DC): Bureau of Justice Statistics; 2016.

[9] World Health Organization. International classification of diseases–6th revision Geneva: World Health Organization; 1948.

[10] World Health Organization. International statistical classification of diseases and related health problems–10th revision Geneva: World Health Organization; 2010 [cited 2017 Sep 1]. http://apps.who.int/classifications/icd10/browse/2010/en.

[11] Centers for Disease Control and Prevention. National Violent Death Reporting System: web coding manual—version 5.1. Atlanta: Centers for Disease Control and Prevention; 2015 [cited 2017 Sep 1]. https://www.cdc.gov/violenceprevention/pdf/nvdrs_web_codingmanual.pdf.

[12] The Counted: people killed by police in the US—about. The Guardian; 2015 [cited 2017 Sep 1]. https://www.theguardian.com/us-news/ng-interactive/2015/jun/01/about-the-counted.

[13] Loftin C, Wiersema B, McDowall D, Dobrin A. Underreporting of justifiable homicides committed by police officers in the United States, 1976–1998. Am J Public Health. 2003;93:1117–21. doi: 10.2105/AJPH.93.7.1117 12835195 10.2105/ajph.93.7.1117 PMC1447919

[14] Barber C, Azrael D, Cohen A, Miller M, Thymes D, Wang DE, et al Homicides by police: comparing counts from the national violent death reporting system, vital statistics, and supplementary homicide reports. Am J Public Health. 2016;106:922–7. doi: 10.2105/AJPH.2016.303074 26985611 10.2105/AJPH.2016.303074 PMC4985110

[15] Feldman JM, Gruskin S, Coull BA, Krieger N. Killed by police: validity of media-based data and misclassification of death certificates in Massachusetts, 2004–2016. Am J Public Health. 2017 8 17 doi: 10.2105/AJPH.2017.303940 28817335 10.2105/AJPH.2017.303940 PMC5607669

[16] Legewie J, Fagan J. Group threat, police officer diversity and the use of police force. New York: Columbia Law School; 2016. Columbia Public Law Research Paper No. 14–512.

[17] Ross CT. A multi-level Bayesian analysis of racial bias in police shootings at the county-level in the United States, 2011–2014. PLoS ONE. 2015;10:e0141854 doi: 10.1371/journal.pone.0141854 26540108 10.1371/journal.pone.0141854 PMC4634878

[18] Krieger N, Chen JT, Waterman PD, Kiang MV, Feldman J. Police killings and police deaths are public health data and can be counted. PLOS Med. 2015;12:e1001915 doi: 10.1371/journal.pmed.1001915 26645383 10.1371/journal.pmed.1001915 PMC4672939

[19] Swaine J, Laugh. Never before named: five people killed by police the world forgot. The Guardian. 2015 Jun 3 [cited 2017 Sep 1]. https://www.theguardian.com/us-news/2015/jun/03/counted-police-killing-victims-unnamed-texas-california.

[20] Jauchem JR. Deaths in custody: are some due to electronic control devices (including TASER devices) or excited delirium? J Forensic Leg Med. 2010;17:1–7. doi: 10.1016/j.jflm.2008.05.011 20083043 10.1016/j.jflm.2008.05.011

[21] National Center for Health Statistics. National Death Index. 2017 [cited 2017 Jun 11]. https://www.cdc.gov/nchs/ndi/index.htm.

[22] National Center for Health Statistics. National Death Index user’s guide. Hyattsville (Maryland): National Center for Health Statistics; 2013.

[23] Bishop YM, Fienberg SE, Holland PW. Discrete multivariate analysis: theory and practice. New York: Springer; 2007.

[24] US Centers for Disease Control and Prevention. Mortality multiple cause files: U.S. data—2015. 2015 [cited 2017 Sep 1]. https://www.cdc.gov/nchs/data_access/VitalStatsOnline.htm.

[25] Tilling K. Capture-recapture methods—useful or misleading? Int J Epidemiol. 2001;30:12–4. doi: 10.1093/ije/30.1.12 11171841 10.1093/ije/30.1.12

[26] Bice D. Medical examiner “threatened” by Clarke over jail deaths. Milwaukee Journal Sentinel. 2016 Dec 2 [cited 2017 Sep 1]. http://www.jsonline.com/story/news/investigations/daniel-bice/2016/12/01/medical-examiner-threatened-clarke-over-jail-deaths/94746392/.

[27] Small J. Keeping death investigations free from pressure. KQED News. 2016 Sep 13 [cited 2017 Sep 1]. https://ww2.kqed.org/news/2016/09/13/keeping-death-investigations-free-from-pressure/.

[28] Simerman J, Mustian J. Coroner reclassifies Henry Glover’s death as homicide in post-Hurricane Katrina police shooting case. The New Orleans Advocate. 2015 Apr 3 [cited 2017 Sep 1]. http://www.theadvocate.com/new_orleans/news/article_c66ed69b-b5f7-564f-aa7a-d0284e3fd48e.html.

[29] Lum K, Ball P. Estimating undocumented homicides with two lists and list dependence. Human Rights Data Analysis Group; 2015 [cited 2017 Sep 1]. https://hrdag.org/wp-content/uploads/2015/07/2015-hrdag-estimating-undoc-homicides.pdf.

[30] United States Census Bureau. 2015 American community survey 5-year estimates. Suitland (Maryland): United States Census Bureau; 2016.

[31] Ingram DD, Franco SJ. 2013 NCHS urban-rural classification scheme for counties. Vital Health Stat 2. 2014;166:1–73. 24776070

[32] US Centers for Disease Control and Prevention. Public Health Law Program: death investigation systems. 2016 [cited 2017 Sep 1]. https://www.cdc.gov/phlp/publications/coroner/death.html.

[33] US Centers for Disease Control and Prevention. Bridged-race population estimates. 2017 [cited 2017 Jun 6]. https://wonder.cdc.gov/bridged-race-population.html.

[34] Miller C, Rainie L, Purcell K, Mitchell A, Rosenstiel T. How people get local news and information in different communities. Washington (DC): Pew Research Center; 2012.

[35] California Department of Justice. Deaths in custody, 2015. Sacramento (CA): Criminal Justice Statistics Center; 2016.

[36] MacKenzie EJ, Hoyt DB, Sacra JC, Jurkovich GJ, Carlini AR, Teitelbaum SD, et al National inventory of hospital trauma centers. JAMA. 2003;289:1515–22. doi: 10.1001/jama.289.12.1515 12672768 10.1001/jama.289.12.1515

[37] Hanzlick R, Goodin J. Mind your manners. Am J Forensic Med Pathol. 1997;18:228–45. doi: 10.1097/00000433-199709000-00002 9290869 10.1097/00000433-199709000-00003

[38] German RR, Fink AK, Heron M, Stewart SL, Johnson CJ, Finch JL, et al The accuracy of cancer mortality statistics based on death certificates in the United States. Cancer Epidemiol. 2011;35:126–31. doi: 10.1016/j.canep.2010.09.005 20952269 10.1016/j.canep.2010.09.005

[39] Foreman KJ, Naghavi M, Ezzati M. Improving the usefulness of US mortality data: new methods for reclassification of underlying cause of death. Popul Health Metr. 2016;14:14 doi: 10.1186/s12963-016-0082-4 27127419 10.1186/s12963-016-0082-4 PMC4848792

[40] Loftin C, McDowall D, Curtis KM, Fetzer MD. The accuracy of Supplementary Homicide Report rates for large U.S. cities. Homicide Stud. 2015;19:6–27. doi: 10.1177/1088767914551984

[41] US Centers for Disease Control and Prevention. National Violent Death Reporting System. 2016 [cited 2017 Sep 1]. https://www.cdc.gov/violenceprevention/nvdrs/.

[42] Attorney General of Texas. Custodial death report. 2016 [cited 2016 Nov 1]. https://oagtx.force.com/cdr/cdrreportdeaths.

[43] State of Tennessee. Public Chapter No. 896. House Bill 2122 (Apr. 27, 2016) [cited 2017 Sep 1]. http://wapp.capitol.tn.gov/apps/Billinfo/default.aspx?BillNumber=HB2122&ga=109.

[44] Feldman JM, Chen JT, Waterman PD, Krieger N. Temporal trends and racial/ethnic inequalities for legal intervention injuries treated in emergency departments: US men and women age 15–34, 2001–2014. J Urban Health. 2016;93:797–807. doi: 10.1007/s11524-016-0076-3 27604614 10.1007/s11524-016-0076-3 PMC5052149

