# Correction: Quantifying underreporting of law-enforcement-related deaths in United States vital statistics and news-media-based data sources: A capture-recapture analysis

**DOI:** 10.1371/journal.pmed.1002449

**Published:** 2017-10-26

**Authors:** Justin M. Feldman, Sofia Gruskin, Brent A. Coull, Nancy Krieger

The fourth sentence in the fourth paragraph of the Discussion section is incorrect. The correct sentence is: Second, Lum and Ball [29], adjusting for potential list dependency but not for agency nonresponse, used the same BJS data to estimate an annual mean of 1,250 deaths in the US, which is higher than our estimate.
